# Supplementary material for: Increasing secular trends in height and obesity in children with type 1 diabetes: JSGIT cohort
Source: PLoS One. 2020 Nov 23;15(11):e0242259. doi: 10.1371/journal.pone.0242259 (PMC7682904; doi:10.1371/journal.pone.0242259)
Supplement: Attachment — Submitted filename: renamed_ca81c.docx [file pone.0242259.s003.docx]

**Responses to the Reviewer**

We thank the reviewers for their valuable comments and questions and for the opportunity to improve our paper.

As requested, we prepared a revised version of our manuscript and hope that we have addressed the concerns of Reviewer #1. Our point-by-point responses to the reviewer’s comments follow, in this letter.

We sincerely thank the reviewers for all of the helpful comments.

**Responses to the comments of reviewer #1**

1. *Line 244: "the increase that we observed in the use of bolus insulin at tea-time suggests that a subset of the patients had excess caloric intake". This is a strong statement without supporting data. Please rephrasing the sentence, at least adding "might" or similar to soften the argument.*

**Response:** We appreciate your insightful comment. We revised manuscript as follows:

“Although we did not assess patients’ diets in our study, the increase that we observed in the use of bolus insulin at tea-time might suggest that a subset of the patients had excess caloric intake.” (line 257)

2. *Table 1 (number of participants). I may consider describing the p-value of the number of participants rather than just dichotomizing p-value (significant vs non-significant).*

**Response:** We appreciate this recommendation to clarify the data in Table 1. The number of participants showed a significantly increased trend (P<0.001), but the proportion of boys did not show no significant trend. We revised Table 1, as follows:

**Table 1. Participants’ characteristics.**

|  | 1995 | 2000 | 2008 | 2013 | *P*-value for the trend^*^ |
| --- | --- | --- | --- | --- | --- |
| Number of Institutions | 37 | 51 | 64 | 68 |  |
| Number of participants (% boys) | 513 (41.7) | 685 (37.4) | 734 (39.9) | 978 (41.3) | <0.001 (0.632) |
| Age, years | 13.4 (2.9) | 13.1 (3.7) | 12.4 (3.6) | 11.9 (3.6) | <0.001 |
| Age at onset, years | 7.2 (3.7) | 7.1 (3.8) | 6.6 (3.7) | 6.7 (3.7) | <0.001 |
| Duration of diabetes, years | 6.2 (3.5) | 5.9 (3.5) | 5.8 (3.5) | 5.1 (3.6) | <0.001 |

The data are presented as mean (standard deviation) or number (%).

*P*-values for the trends as determined by Cochran–Armitage or Jonckheere–Terpstra tests.

3. *Table 2. Why are we missing p-for-trend in total daily insulin use? Also, how did the authors calculate p-for-trend in proportion of patients using insulin analogue? (e.g., at basal, did they calculate it using the data from 2008 and 2013? or did they also include "0" value in 1995 and 2000?) I would suggest adding the detailed explanation at least in the footnote of this table.*

**Response:** We appreciate your valuable suggestions to clarify these details.

Total daily insulin dose showed no significant trend (P=0.992). Therefore, we determined P values <0.001 by the Mann–Whitney U test and compared the results with those in 1995. We revised the discussion of the findings in Table 2, as follows:

Because “in Japan, rapid-acting insulin analogues were introduced in 2000, and long-acting insulin analogues were introduced in 2003” (line 250), no patients used insulin analogues or insulin analogues as a bolus or as basal therapy. P-values were calculated using the data from 1995 to 2013, including a "0" value in 1995 or 2000. We added the following detailed explanation in the footnote to Table 1: “0, no patients used insulin analogues or insulin analogues as a bolus or as basal therapy.”, and “P-values for the trends as determined by the Cochran–Armitage or Jonckheere–Terpstra tests using data the from 1995 to 2013.”

**Table 2. Secular changes in height SDSs and the prevalence of overweight and obesity in Japanese children with type 1 diabetes mellitus.**

| Cohort | 1995 | 2000 | 2008 | 2013 | *P*-value for the trend^*^ |
| --- | --- | --- | --- | --- | --- |
| Number, (% male) | 513 (41.7) | 685 (37.4) | 734 (39.9) | 978 (41.3) |  |
| HbA1c, % (SD) | 9.4 (2.0) | 8.4 (1.6) | 7.8 (1.1) | 8.0 (1.2) | <0.001 |
| HbA1c, mmol/mol (SD) | 78.9 (22.4) | 68.3 (18.0) | 61.4 (12.5) | 64.1 (13.3) | <0.001 |
| Incidence of severe hypoglycaemic events per 100 patient-years (95% CI) | No data | 21.0 (8.5–33.6) | 7.4 (3.1–11.6) | 4.8 (3.2–6.3) | <0.001 |
| Proportion of patients using insulin analogues, % | 0 | 14.6 | 94.7 | 98.6 | <0.001 |
| As bolus, % | 0 | 14.6 | 88.6 | 87.2 | <0.001 |
| As basal, % | 0 | 0 | 94.7 | 96.5 | <0.001 |
| Proportion of patients using bolus insulin at tea-time, % | No data | 1.72 | 19.7 | 26.3 | <0.001 |
| Total daily insulin dose, units/kg/day (SD) | 1.01 (0.32) | 1.08 (0.35)^†^ | 1.09 (0.33)^‡^ | 1.02 (0.37) | 0.992 |
| Height SDS, mean (SD) | −0.22 (1.07) | −0.21 (1.24) | +0.04 (1.08) | +0.17 (1.02) | <0.001 |
| BMI >85th percentile, (%) | 59 (11.50) | 114 (16.64) | 136 (18.53) | 196 (20.04) | <0.001 |
| BMI percentile, mean (SD) | 52.1 (25.3) | 58.1 (25.8) | 58.2 (26.0) | 58.7 (26.1) | <0.001 |
| BMI SDS, mean (SD) | +0.02 (1.18) | +0.21 (1.02) | +0.25 (0.93) | +0.28 (0.90) | <0.001 |
| Obese (%) | 11 (2.14) | 25 (3.65) | 48 (5.18) | 50 (5.11) | 0.004 |
| Overweight (%) | 48 (9.36) | 89 (12.99) | 98 (13.35) | 146 (14.93) | 0.005 |

The data are presented as mean (SD) or number (%).

^*^*P*-values for the trends as determined by the Cochran–Armitage or Jonckheere–Terpstra tests using data from 1995 to 2013.

^†^*P*=0.001, ^‡^*P*<0.001 vs. 1995 as determined by the Mann–Whitney U test.

SD, standard deviation; 95% CI, 95% confidence interval; BMI, body mass index; SDS, standard deviation score; obese, BMI ≥95th percentile; overweight, BMI in the 85th–94th percentile; 0, no patients used insulin analogues or insulin analogues as a bolus or as basal therapy.

4. *Figure 2. I appreciate the authors creating this nice figure. Please fix the x-label for c) SDS because it doesn't seem be in the appropriate position.*

**Response:** We appreciate your helpful comment. We moved the x-axis label to the appropriate position, as follows:

We hope we have addressed the main points raised by you and the reviewers. Once again, we are extremely grateful for your and the reviewers’ insightful suggestions and hope that the manuscript is now suitable for publication in *PLoS One.*

Sincerely,

Authors
